# Predicting treatment failure in patients with community acquired pneumonia: a case-control study

**DOI:** 10.1186/1465-9921-15-75

**Published:** 2014-07-05

**Authors:** Ignacio Martin-Loeches, Xavier Valles, Rosario Menendez, Oriol Sibila, Beatriz Montull, Catia Cilloniz, Antonio Artigas, Antoni Torres

**Affiliations:** 1 Critical Care Center, Sabadell Hospital, CIBER Enfermedades Respiratorias, Parc Tauli University Institute, Sabadell, Spain; 2 Hospital Clinic, IDIBAPS, Universitat de Barcelona, Servei de Pneumologia, Institut del Torax, Barcelona, Spain; 3 Hospital Universitari i Politècnic La Fe, Servicio de Neumología, Valencia, Spain; 4 Department of Pneumology, Hospital de la Santa Creu i Sant Pau, Barcelona, Spain; 5 Hospital Clinic, IDIBAPS, Universitat de Barcelona, CIBER enfermedades respiratorias, Servei de Pneumologia, Institut del Torax, Villarroel 170, 08036 Barcelona, Spain

**Keywords:** Treatment failure, Cytokines, Pneumonia, Community acquired pneumonia

## Abstract

**Introduction:**

Treatment failure in community-acquired-pneumonia (CAP) patients is associated with a high mortality rate, and therefore are a matter of great concern in clinical management. Those patients have increased mortality and are a target population for randomized clinical trials.

**Methods:**

A case–control study was performed in patients with CAP (non-failure cases vs. failure cases, discriminating by late and early failure). CRP, PCT, interleukin 1, 6, 8 and 10 and TNF were determined at days 1 and 3 of hospitalization.

**Results:**

A total of 253 patients were included in this study where 83 patients presented treatment failure. Of these, 40 (48.2%) had early failure. A discriminative effect was found for a higher CURB-65 score among late failure patients (p = 0.004). A significant increase on day 1 of hospitalization in CRP (p < 0.001), PCT (p = 0.004), IL-6 (p < 0.001) and IL-8 (p = 0.02), and a decrease in IL-1 (p = 0.06) in patients with failure was observed compared with patients without failure. On day 3, only the increase in CRP (p < 0.001), PCT (p = 0.007) and IL-6 (p < 0.001) remained significant. Independent predictors for early failure were higher IL-6 levels on day 1 (OR = 1.78, IC = 1.2-2.6) and pleural effusion (OR = 2.25, IC = 1.0-5.3), and for late failure, higher PCT levels on day 3 (OR = 1.60, IC = 1.0-2.5), CURB-65 score ≥ 3 (OR = 1.43, IC = 1.0-2.0), and multilobar involvement (OR = 4.50, IC = 2.1-9.9).

**Conclusions:**

There was a good correlation of IL-6 levels and CAP failure and IL-6 & PCT with late CAP failure. Pleural effusion and multilobar involvement were simple clinical predictors of early and late failure, respectively.

**Trial registration:**

IRB Register: http://2009/5451.

## Introduction

Community-acquired pneumonia (CAP) is a major health problem worldwide. Clinically, CAP exhibits enormous variety in the severity of presentation, from septic shock at one end of the spectrum to almost asymptomatic disease at the other [1]. Treatment failure is a matter of great concern in the management of CAP. The reported incidence of treatment failure among hospitalized patients with CAP ranges from 2.4% to 31% for early failure and from 3.9% to 11% for late failure [2].

It has been recognized that an excessive systemic proinflammatory response in patients with sepsis and severe CAP is associated with deleterious effects and a worse prognosis [3]. Conversely, an exaggerated anti-inflammatory response conducted by some grade of impaired immune response may have a negative effect on clinical resolution [4]. Among the cytokines involved in the inflammatory response, we can include interleukin 10 (IL-10) as a critical anti-inflammatory cytokine that attenuates inflammatory responses in macrophages and T cells, tumor necrosis factor (TNF-α), interleukin 1 (IL-1), interleukin 6 (IL-6) and interleukin 8 (IL-8) [5]. Some inflammation markers, such as C-reactive protein (CRP) and procalcitonin (PCT), have been recognized for some time in clinical management, [6]. Therefore, besides clinical factors, cytokine levels can predict CAP-failure and should be included to determine those patients with CAP who are most at risk of developing treatment failure. Early prediction of CAP-failure is a critical step toward better management and improvement of survival. In this study, we aimed to determine clinical and cytokine-level predictors of CAP-failure, including the different types of failure (early and late). The aim of this research is to ascertain the parameters, including cytokine levels, for predicting CAP failure in general, and more specifically, late and early failure.

## Methods

### Ethical issues

This study was approved by the Ethics Committee of Hospital Clinic, Barcelona, Spain (IRB Register: 2009/5451) and patients or their relatives signed informed consent.

### Patients and methods

A case–control study in patients with CAP was conducted by the Pneumology Department in Hospital Clínic, Barcelona, Spain and Hospital de la Fe, Valencia, Spain. Cases were considered to be patients with CAP who developed treatment failure and controls were CAP patients who did not develop failure after a follow-up until they were discharged. Patients with CAP (failure and non-failure) were enrolled between January 2002 and December 2003. For each case, 1 to 2 controls were included and matched by age and time of hospitalization (hospitalized during the same week).

The diagnosis of pneumonia was based on the presence of acute onset of signs and symptoms suggesting lower respiratory tract infection on admission, and radiographic evidence of a pulmonary infiltrate that had no other known cause [7]. CAP and Health Care—Associated Pneumonia (HCAP) patients were included in the present study. Exclusion criteria were patients with tuberculosis, obstructive pneumonia caused by neoplasia or pneumonia as a terminal event of chronic and progressive disease, HIV infection, neoplasia, those taking cytotoxic drugs or long-term oral steroid therapy, such as daily dose of 20 mg of prednisolone or the equivalent for >2 weeks and patients with a previous admission within the previous 15 days.

### Data collection

Patients with CAP (failure and non-failure) were enrolled over a 24 month period. All CAP-failure without exclusion criteria were included, and 1 to 2 non-failure CAP hospitalized within the same period of admission (within the same week) were included as controls.

The following data were recorded from CAP patients using a standard questionnaire: age, gender, smoking history, alcohol abuse (> 80 g/day) and comorbidities (chronic obstructive pulmonary disease (COPD), cardiac, liver, renal, digestive or central nervous system diseases). The initial severity was assessed using the pneumonia severity index (PSI) and CURB 65 (confusion, urea, respiratory rate, blood pressure and age) score [8].

Early treatment failure was defined, as clinical deterioration within 72 h of treatment, as indicated by development of shock, need for invasive mechanical ventilation, or death. Late treatment failure was defined as radiographic progression (increase > 50% of pulmonary infiltrates compared to baseline), persistence of severe respiratory failure (PaO_2_/FiO_2_ < 200, with respiratory rate > 30 min-1 in non-intubated patients), development of shock, need for invasive mechanical ventilation, or death between 72 and 96 h after start of treatment [9,10]. Severe sepsis and septic shock were defined following the criteria of the American College of Chest Physicians and the Society of Critical Care Medicine [11]. The indications for mechanical ventilation and/or inotropic support were left to the discretion of the attending physicians.

### Measurements of serum cytokines and laboratory procedures

Blood samples were drawn from all patients on enrolment in the study, immediately at the moment of pneumonia diagnosis, and at day three after admission. Blood was centrifuged, coded, and serum aliquoted and frozen at -80°C until subsequent analysis. IL-10, TNF-α, IL-1, IL-8 and IL-6 protein levels were measured blinded to the groups, using a commercial enzyme immunoassay technique (BioSource, Nivelles, Belgium). The limit of detection was 1 pg/mL. CRP and PCT were measured using standard procedures. Cytokine measurements were carried out on the day of hospitalization (day 1) and at day 3 of hospitalization. Microbiological diagnostic were carried out using routine procedures.

### Statistical analysis and data management

Statistical analysis was performed using the SPSS 17.0 and STATA 10.0 software programs. Results are presented as mean, standard deviation (SD), median, frequency or percentage, as required. Continuous variables were analyzed using the *t* test, whereas categorical variables were analyzed using the χ^2^ test. Non-parametric tests (Mann–Whitney U, Kruskal-Wallis or Fisher test) were used when necessary. The association was adjusted using multivariate logistic regression and multinomial (polytomous) logistic regression when stratifying cases by early and late failure [12]. ROC analysis was performed for each cytokine level regarding CAP failure, early CAP failure and late CAP failure. CRP levels were used as a baseline for comparisons. Cut-offs of maximum sensitivity/specificity were determined for each cytokine level and different outcomes (failure, early failure and late failure), at day 1 and day 3 of hospitalization. Cytokine levels were categorized and analyzed in equivalent quartiles. Effects were calculated as odds ratios (OR) with their corresponding 95% confidence intervals (CI). A p-value of 0.05 was considered significant for the univariate analysis, and variables with p < 0.1 were considered in the logistic regression model. Early failure cases were dropped when considering cytokine levels at day 3 of admission.

## Results

### General description

A total of 253 patients with CAP were included in this study (mean [SD] age, 69.1 [16.1] years; range, 19–91 years): Briefly, the distribution of subjects of CURB 65 classes among CAP patients was: 0-I, n = 102 (40.3%); II-III, n = 117 (46.1%); IV-V, n = 34 (13.4%). Septic shock was present in 31 patients (12.8%). The main demographic characteristics, comorbid conditions and severity are shown in Table 1. Eighty-three patients (32.9%) developed treatment failure, of which 40 (48.2%) had early failure. Table 1 shows the general results and univariate analysis of CAP and CAP failure.

**Table 1:** General description and univariate analysis of variables under study

**Variable (N, %)**	**CAP non-failure N = 170**	**Early failure N = 40**	p value**	**Late failure N = 43**	p value***
Age, mean (SD)	67.2 (17.6)	67.4 (18.5)	0.8	69.0 (15.8)	0.8
Gender, male	116 (68.6)	26 (65)	0.6	24 (55.8)	0.1
Smoking habit	31 (18.3)	8 (20)	0.8	13 (30.2)	0.08
Alcohol intake	17 (10.1)	4 (10)	0.9	5 (11.6)	0.7
Pulmonary diseases	66 (39.1)	18 (45)	0.4	23 (53.3)	0.08
COPD	31 (18.3)	6 (15)	0.6	7 (16.3)	0.8
Previous Antibiotic therapy	61 (36.1)	19 (47.5)	0.1	11 (25.6)	0.2
Previous hospitalization	33 (19.5)	7 (17.5)	0.8	7 (16.3)	0.6
Previous CAP	48 (28.4)	8 (20)	0.3	9 (20.9)	0.3
Comorbidity (all)	93 (54.7)	15 (37.5)	0.05	24 (55.8)	0.9
*Neurological*	47 (27.7)	9 (22.5)	0.5	13 (30.2)	0.7
Cirrhosis	5 (2.9)	1 (2.5)	0.8	2 (4.7)	0.6
Cardiac insufficiency	33 (19.4)	4 (10.0)	0.2	11 (25.6)	0.4
Chronic Renal failure	14 (8.3)	1 (2.5)	0.2	1 (2.3)	0.2
Diabetes Mellitus	39 (22.9)	7 (17.5)	0.5	7 (16.3)	0.3
*Digestive*	34 (20.0)	8 (18.6)	0.8	7 (17.5)	0.7
Multilobar involvement	34 (20.0)	23 (53.5)	< 0.001	14 (35.0)	0.04
ICU admission	5 (3)	6 (15)	0.002	24 (55.8)	< 0.001
PSI IV-V	108 (63.9)	22 (55)	0.3	34 (79.1)	0.06
CURB 65 ≥ 3	50 (29.4)	14 (35.0)	0.6	22 (51.2)	0.004
Pleural effusion	24 (14.1)	13 (32.5)	0.006	9 (20.9)	0.3
Mortality	3 (1.8)*	13 (32.5)	< 0.001	14 (32.6)	< 0.001
Length of hospital stay, mean (SD)	8.1 (5.7)	17.5 (10.5)	< 0.001	17.1 (17.1)	< 0.001
Pneumococcal pneumonia	28 (16.5)	8 (20.0)	0.6	8 (18.6)	0.7

*Patients included in this category; mortality was not directly attributed to the pneumonia and occurred > 96 h, after admission.

**p value comparing early failure cases with control group (non-failure CAP patients).

***p value comparing late failure cases with control group (non-failure CAP patients).

Microbiological documentation of CAP was achieved in 106 patients (41.9%). *Streptococcus pneumoniae* was the most common pathogen isolated (17.8%), followed by *Legionella pneumophila* (5.1%) and *Staphylococcus aureus* (3.9%). A total of 46.9% of the patients received third generation cephalosporin and a macrolide, 23.2% quinolone alone and 8.7% third generation cephalosporin and quinolone as first line treatment. Significant differences were found between patients with and without treatment failure. Patients with treatment failure presented higher severity index or PSI scores (mean [SD], 109.6 [42.2] vs. 97.8 [36.6]; p = 0.02), higher prevalence of pleural effusion (26.5% vs. 14.1%; p = 0.006), and ICU admission (36% vs. 3%; p < 0.001) with multilobar involvement (52.1% vs. 47.2%; p < 0.001) than those without treatment failure. Length of hospital stay was higher in patients who presented treatment failure (mean [SD], 17.1 [14.1] days vs. 8.1 [5.7] days; p < 0.001). Thirty-one patients died (12.3%): 28 of 83 with treatment failure (33%) and 3 of 170 without treatment failure (1.8%) (p < 0.001). Data are summarized in Table 1.

### Cytokine levels and CAP failure

**Figure 1:**
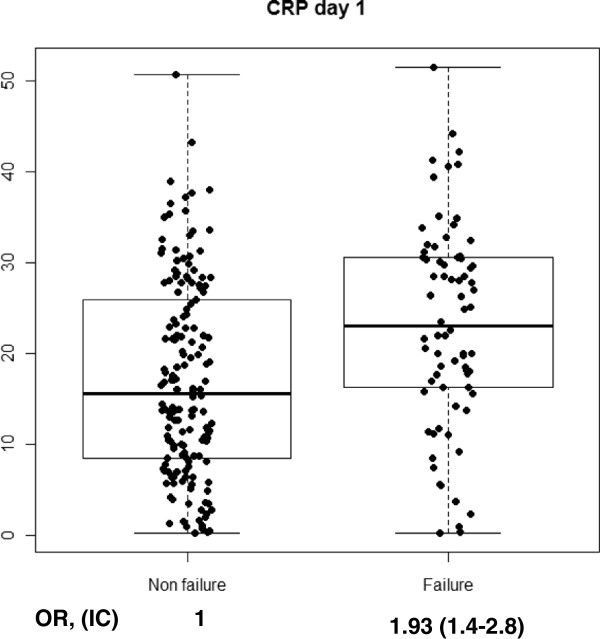
Box-plot and OR for trend for treatment failure and CRP at day 1 of hospitalization.

**Figure 2:**
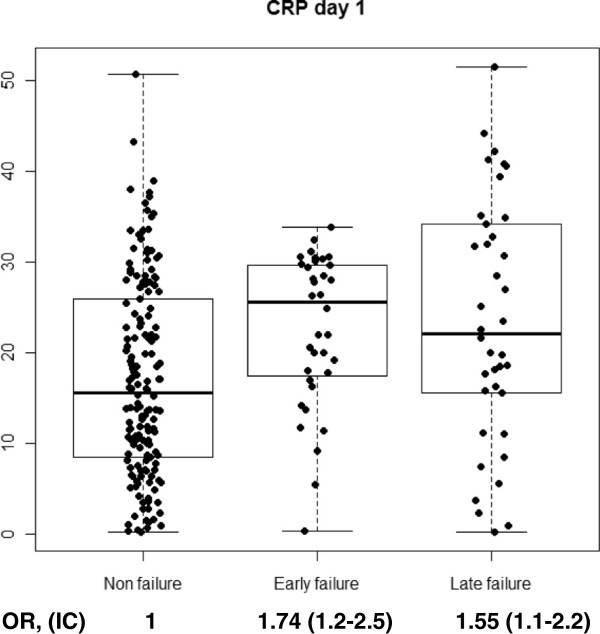
Box-plot and OR for trend for treatment failure and IL-6 at day 1 of hospitalization.

**Figure 3:**
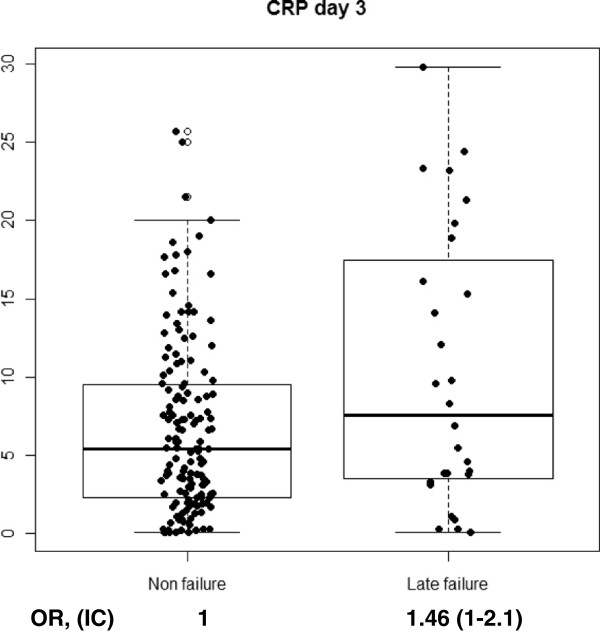
Box-plot and OR for trend for treatment failure and IL-8 at day 1 of hospitalization.

**Figure 4:**
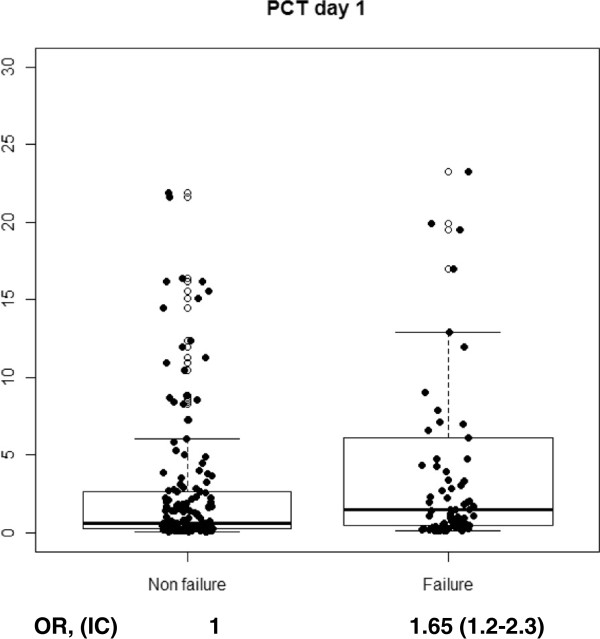
Box-plot and OR for trend for treatment failure and PCT at day 1 of hospitalization.

With regard to cytokine levels, when comparing CAP vs. CAP failure, we found a significant increase on day 1 of hospitalization in CRP (p < 0.001), PCT (p = 0.004), IL-6 (p < 0.001) and IL-8 (p = 0.02). When stratifying CAP-failure cases by early failure or late failure, we found a discriminating effect of PCT for late failure on day 1 (p = 0.005), while it remained non-significant among patients with early failure (p = 0.3). This was also observed, though less marked, in IL-8 on day 1 (p = 0.06) and a protective effect of IL-1 against late failure was found only on day 1 (p = 0.04). On day 3, CRP levels (p = 0.05), IL-6 levels (p = 0.007) and PCT levels (p = 0.001), were significantly higher among patients that developed late failure compared to non-failure cases. No significant increases or decreases were observed in IL-10 or TNF. Figures 1, 2, 3 and 4 show the box-plots and OR comparing CAP vs. CAP failure and CRP, Il-6, IL-8 and PCT on day 1 of hospitalization. Supplementary material (Additional file 1: Figure S1–S10) shows the box-plots and OR for trend for each outcome and cytokine level and day of hospitalization. No measurable predicting effect was observed when considering the coefficient of variation between day 1 and day 3 in cases with treatment failure.

To determine the usefulness of those parameters for predicting CAP failure, we performed ROC (area under the curve, AUC) analysis of significant cytokine levels (CRP, PCT, IL-1, IL-6 and IL-8) (Tables 2, 3 and 4). Taking CRP AUC analysis as a reference, we found that IL-6 showed better performance at predicting CAP failure on day 1. This difference was also observed when stratifying the analysis into early and late failure (Table 4). In addition, we found a discriminating effect of PCT levels compared to CRP in the CAP late-failure group, with a better sensitivity/specificity ratio compared to CRP levels on day 1 and day 3 (Table 3).

**Table 2:** AUC analysis and sensitivity and specificity of Cytokine levels and CAP vs CAP failure

	**Day 1**					
	**AUC**	**95% IC**	**p**	**Cut-off**	**Sensitivity**	**Specificity**
CRP	0.66	*(0.43-0.59)*		19.7	62.2	61.7
PCT	0.62	*(0.54-0.69)*	0.4	1.21	56.8	56.8
Interleukin 1	0.41	*(0.33-0.50)*	< 0.001	159	43.2	42.0
Interleukin 6	0.70	*(0.63-0.77)*	0.2	124	64.9	62.7
Interleukin 8	0.61	*(0.53-0.70)*	0.8	11	56.8	61.7

**Table 3:** AUC analysis and sensitivity and specificity of Cytokine levels and CAP vs Early failure

	**Day 1**					
	**AUC**	**95% IC**	**p**	**Cut-off**	**Sensitivity**	**Specificity**
CRP	0.67	*(0.58-0.76)*		20.2	61.1	63.0
PCT	0.56	*(0.47-0.65)*	0.02	2.69	55.6	55.6
Interleukin 1	0.43	*(0.32-0.54)*	0.002	102.0	44.4	45.0
Interleukin 6	0.72	*(0.64-0.81)*	0.3	142.0	69.4	65.8
Interleukin 8	0.60	*(0.50-0.71)*	0.3	11.0	55.6	55.9

**Table 4:** AUC analysis and sensitivity and specificity of Cytokine levels and CAP vs Late failure

		**CRP**	**PCT**	**Interleukin 1**	**Interleukin 6**	**Interleukin 8**
Day 1	AUC	0.64	0.67	0.40	0.68	0.62
	95% IC	*(0.53-0.75)*	*(0.57-0.77)*	*(0.29-0.51)*	*(0.58-0.79)*	*(0.51-0.74)*
	p		0.4	< 0.001	0.2	0.4
	Cut-off	18.6	1.4	148.0	93.0	11.0
	Sensitivity	60.6	60.5	39.5	57.9	57.9
	Specificity	59.3	60.5	42.0	57.1	55.9
Day3	AUC	0.60	0.69	0.41	0.66	0.52
	95% IC	*(0.47-0.73)*	*(0.59-0.79)*	*(0.28-0.54)*	*(0.54-0.78)*	*(0.39-0.65)*
	p		0.1	< 0.001	0.5	0.008
	Cut-off	5.9	0.47	112.0	36.0	10.0
	Sensitivity	53.6	80.7	46.4	64.3	50.0
	Specificity	53.1	61.9	47.6	61.9	52.1

### Predicting CAP failure

A logistic regression model was applied to all clinical and cytokine levels included in this study to determine independent predictive factors for CAP failure, including early and late failure. A final model to predict CAP failure includes higher IL-6 levels on day 1, CURB 65 score ≥ 3, pleural effusion and multilobar involvement. Regarding early failure, significant and independent predictors were higher IL-6 levels on day 1 and pleural effusion, and for late failure, higher PCT levels on day 3, CURB 65 score ≥ 3, and multilobar involvement. Table 5 shows raw and adjusted analysis of the variables included in the final logistic regression model.

**Table 5:** Univariate (crude) and multivariate (adjusted) analysis of the variables included in the final predictor model for CAP failure, early failure and late failure

		***Univariate analysis***		***Multivariate (Adjusted)***	
		**OR**	**95% IC**	**OR**	**95% IC**
CURB 65 score ≥ 3	*CAP failure*	1.31	*(1.1-1.6)*	1.16	*(0.9-1.5)*
	*Early*	1.08	*(0.8-1.4)*	0.96	*(0.7-1.3)*
	*Late*	1.59	*(1.2-2.1)*	1.43	*(1.0-2.0)*
Multilobar involvement	*CAP failure*	3.21	*(1.8-5.7)*	2.91	*(1.5-5.5)*
	*Early*	2.15	*(1.0-4.6)*	1.79	*(0.8-4.1)*
	*Late*	4.60	*(2.3-9.3)*	4.50	*(2.1-9.9)*
Pleural effusion	*CAP failure*	2.19	*(1.1-4.2)*	1.85	*(0.9-3.8)*
	*Early*	2.93	*(1.3-6.5)*	2.25	*(1.0-5.3)*
	*Late*	1.61	*(0.7-3.8)*	1.40	*(0.5-3.7)*
Interleukin 6 level day 1	*CAP failure*	1.78	*(1.4-2.3)*	1.60	*(1.2-2.1)*
	*Early*	1.85	*(1.3-2.6)*	1.78	*(1.2-2.6)*
	*Late*	1.72	*(1.2-2.4)*	1.42	*(1.0-2.1)*
PCT level day 1	*CAP failure*	1.38	*(1.1-1.8)*	1.10	*(0.8-1.5)*
	*Early*	1.19	*(0.9-1.6)*	0.98	*(0.7-1.4)*
	*Late*	1.61	*(1.2-2.3)*	1.22	*(0.8-1.8)*
Interleukin level day 3	*CAP failure*	----	*----*	----	*----*
	*Early*	----	*----*	----	*----*
	*Late*	1.71	*(1.3-3.0)*	1.37	*(0.9-2.1)*
PCT level day 3	*CAP failure*	----	*----*	----	*----*
	*Early*	----	*----*	----	*----*
	*Late*	2.01	*(1.2-2.5)*	1.60	*(1.0-2.5)*

## Discussion

We determined a set of clinical and biological factors that can be used to predict CAP failure and to discriminate those patients at risk of developing early (IL-6) and late (IL-6 and PCT) failure to improve prevention measures and clinical management. It is important to remark that, in some conditions, clinical symptoms are not accurate enough and clinicians must look for biological markers. In consequence, proper follow-up using specific biological markers should help to discriminate early and late failure and better monitoring of patients at risk.

Early failure has been commonly used as a surrogate for progressive pneumonia with clinical deterioration within the first 72 h after hospital admission [13]. It is in this phase that the patient is more vulnerable to serious deterioration and often requires advance support treatment based on vasopressors and/or mechanical ventilation. In our study, IL-6 showed the best correlation in predicting early CAP failure (better than the widely used CRP). It is important to remark that blood levels of CRP are primarily regulated by the production of IL-6 [14]. In clinical practice the use of CRP can be seen as a surrogate marker for levels of IL-6 [15]. Furthermore, the presence of elevated levels of IL-6 has a good correlation with not only early but also late failure on day 1 and 3 Unfortunately, IL-6 is not used in clinical practice and merits further discussion. IL-6 is a pleiotropic cytokine and its function is yet to be fully determined [16]. IL-6 is a marker of severity and prognosis of severe infections, although their causal relationship remains unclear. IL-6 can be secreted in response to specific microbial molecules that induce intracellular signaling cascades that give rise to inflammatory cytokine production [17]. We also found that IL-6 is a better marker for detecting treatment than PCT. However, PCT may be more valuable for discriminating late failure at an earlier phase than IL-6 and CRP and, therefore, defining a subset of patients who deserve close monitoring. In addition to these results, we show the AUC analysis of the different cytokines considered and the cut-off for sensitivity and sensibility, to explore their usefulness as treatment failure predictors in future prospective studies. No relevant association was found for IL-10 and TNF levels and CAP failure.

In terms of the clinical factors under study, we found that multilobar involvement associated not only with general with CAP failure, but also specifically with late treatment failure. Other authors have investigated independent factors associated with early deaths in CAP and have shown that age, abnormal mental status, multilobar pneumonia, shock, bacteremia, and inadequate empiric antibiotic therapy were predictors of death within 48 hours [18]. Knowing predictors of treatment failure is important because this outcome parameter may assist further studies in understanding the benefits of new antibacterial or co-adjuvant therapies without including a large population, which would be needed if mortality was the outcome chosen [19].

To our knowledge, this is the first study in which the kinetics of biomarkers have been investigated to detect treatment failure using a case–control design. In this sense, it is remarkably different than previous observational studies. Our findings reinforce results from previous studies [10], [1]. Nevertheless, this study has a more suitable design, which may sustain implementation of scientific results in clinical practice. In the case of treatment failure in CAP, we have followed recommendations: 1-Starting with cross sectional studies, 2-Confirming these results with a case–control and 3-The final step would be to carry out proper cohort studies.

This study has some limitations that should be mentioned. The number of critically ill patients admitted is low and our results need to be validated in a cohort of patients admitted to ICU in order to minimize selection bias. One of the limitations of this study as well as several other studies of biomarkers of severity or failure is that although the mean values of certain cytokines are statistically different from patients with failure vs patients without failure, the wide range of values for particular cytokines make it difficult to use the value of a single patient to predict clinical outcomes. Although samples were not taken from patients on a daily basis, we chose to obtain samples at day 1 and 3 in order to implement our study design in current clinical practice, and some patients without treatment failure were discharged before day three (n = 24). However, when excluding those patients from the general analysis, results and conclusions showed no significant changes.

In summary, patients with treatment failure presented higher levels of IL-6 on admission to hospital and a CURB 65 score of 3 or more. Interestingly, high levels of IL-6 had a high predictive value for early and late failure on days 1 and for late failure on day 3, showing that IL-6 is a good marker for progression to treatment failure, and PCT may have a discriminative effect for predicting late failure. In conclusion, a combination of IL-6, PCT and CURB 65 score could provide a new tool for predicting failure and early and late failure. Pleural effusion and multilobar involvement were simple clinical predictors of early and late failure, respectively.

## Competing interests

The authors declare that they have no competing interests.

## Authors’ contributions

IM-L, XV and AT assisted in the design of the study, coordinated patient recruitment, analyzed and interpreted the data, and assisted in writing the paper. CE, OS, CC, RM and BM made important contributions to the acquisition and analysis of data. IML, XV, AA and AT were involved in revising the manuscript critically for important intellectual content. AT, XV and IML made substantial contributions to the concept, design, analysis and interpretation of data and revised the final manuscript version. All authors read and approved the final manuscript. AT acted as guarantor of/person responsible for the entire manuscript.

## Supplementary Material

Additional file 1: Figure S1 Box-plot and OR for trend for early and late treatment failure and CRP at day 1 of hospitalization. **Figure S2.** Box-plot and OR for trend for late treatment failure and CRP at day 3 of hospitalization. **Figure S3.** Box-plot and OR for trend for early and late treatment failure and PCT at day 1 of hospitalization. **Figure S4.** Box-plot and OR for trend for late treatment failure and PCT at day 3 of hospitalization. **Figure S5.** Box-plot and OR for trend for treatment failure and IL-1 at day 1 of hospitalization. **Figure S6.** Box-plot and OR for trend for early and late treatment failure and IL-1 at day 1 of hospitalization. **Figure S7.** Box-plot and OR for trend for late treatment failure and IL-1 at day 3 of hospitalization. **Figure S8.** Box-plot and OR for trend for early and late treatment failure and IL-6 at day 1 of hospitalization. **Figure S9.** Box-plot and OR for trend for late treatment failure and IL-6 at day 3 of hospitalization. **Figure S10.** Box-plot and OR for trend for early and late treatment failure and IL-8 at day 1 of hospitalization. Click here for file
