# Supplementary material for: Predicting treatment failure in patients with community acquired pneumonia: a case-control study
Source: Respir Res. 2014 Jul 5;15(1):75. doi: 10.1186/1465-9921-15-75 (PMC4099489; doi:10.1186/1465-9921-15-75)
Supplement: Additional file 1: Figure S1 — Box-plot and OR for trend for early and late treatment failure and CRP at day 1 of hospitalization. Figure S2. Box-plot and OR for trend for late treatment failure and CRP at day 3 of hospitalization. Figure S3. Box-plot and OR for trend for early and late treatment failure and PCT at day 1 of hospitalization. Figure S4. Box-plot and OR for trend for late treatment failure and PCT at day 3 of hospitalization. Figure S5. Box-plot and OR for trend for treatment failure and IL-1 at day 1 of hospitalization. Figure S6. Box-plot and OR for trend for early and late treatment failure and IL-1 at day 1 of hospitalization. Figure S7. Box-plot and OR for trend for late treatment failure and IL-1 at day 3 of hospitalization. Figure S8. Box-plot and OR for trend for early and late treatment failure and IL-6 at day 1 of hospitalization. Figure S9. Box-plot and OR for trend for late treatment failure and IL-6 at day 3 of hospitalization. Figure S10. Box-plot and OR for trend for early and late treatment failure and IL-8 at day 1 of hospitalization. [file 1465-9921-15-75-S1.ppt]

## Slide 1
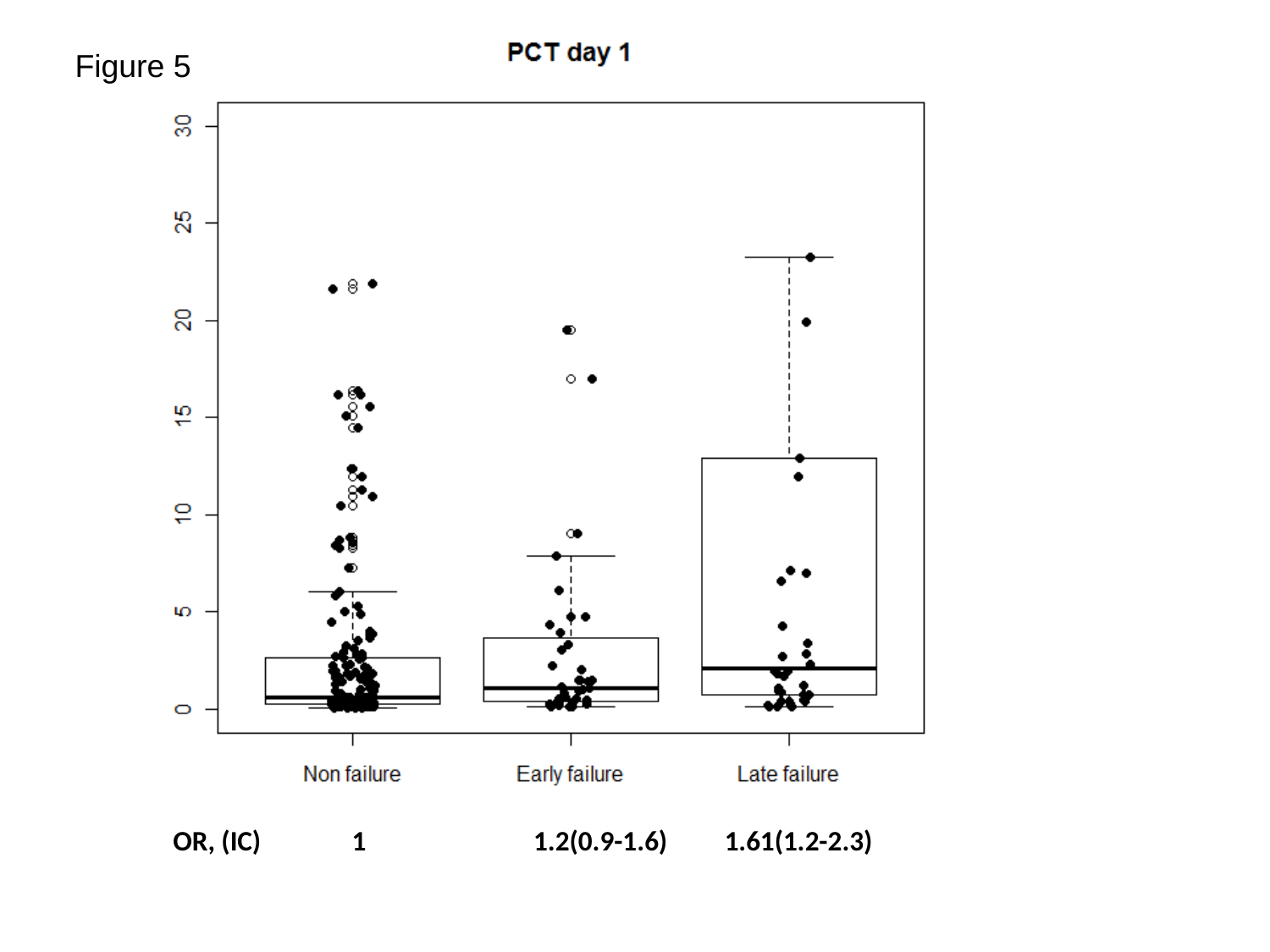

Figure 5
OR, (IC)
 1
1.2(0.9-1.6) 1.61(1.2-2.3)

## Slide 2
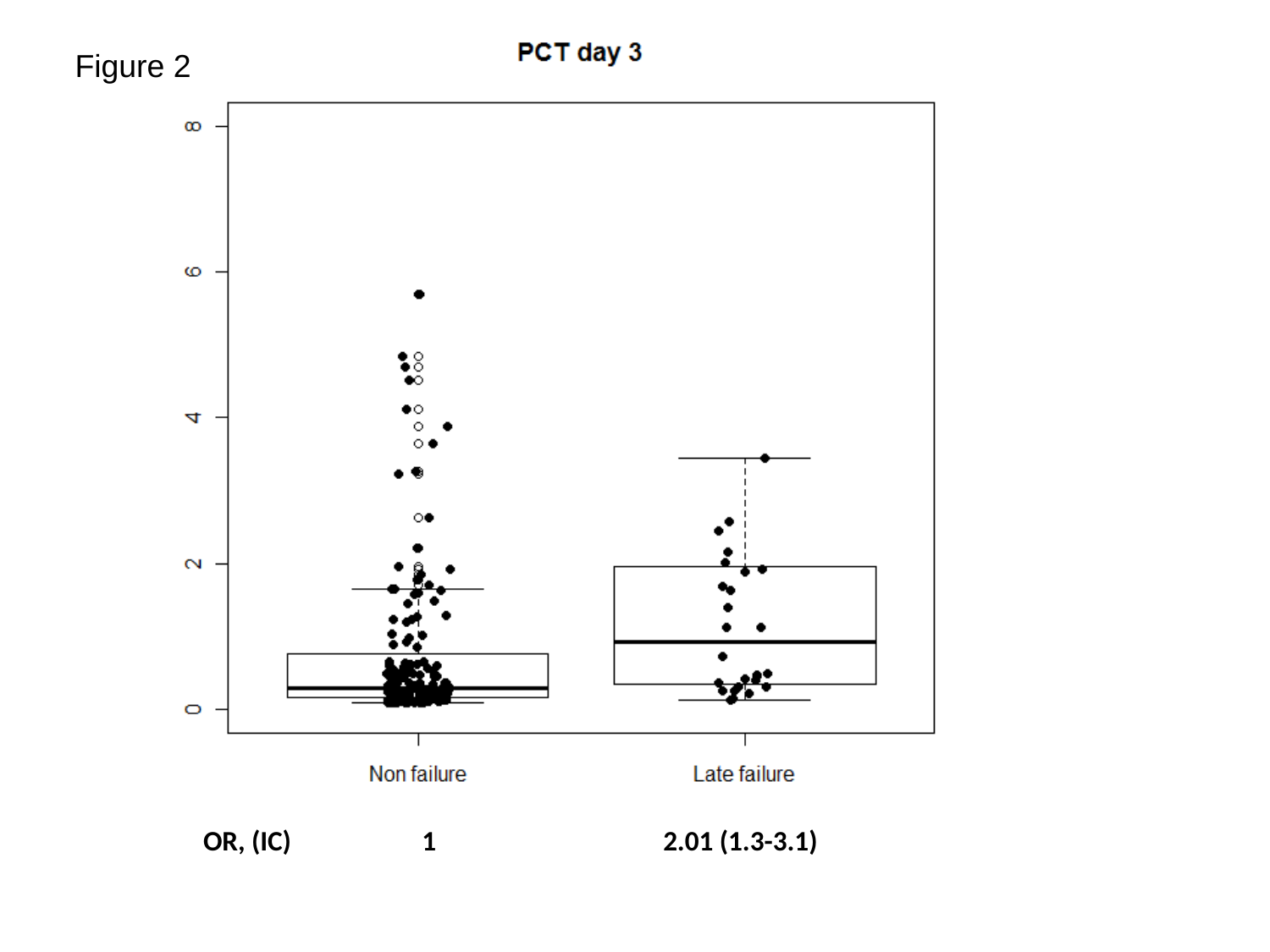

Figure 2
OR, (IC)
 1
2.01 (1.3-3.1)

## Slide 3
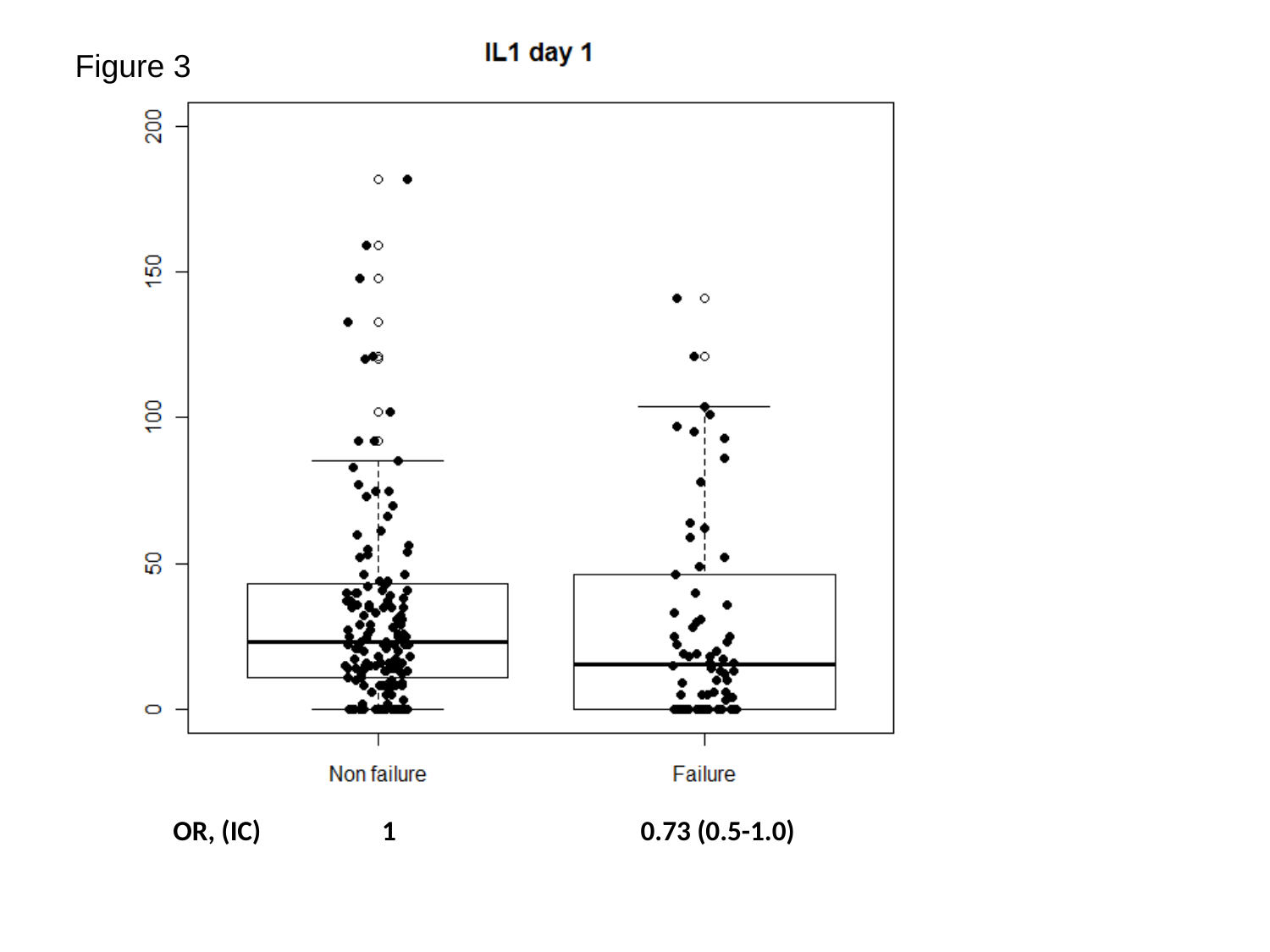

Figure 3
OR, (IC)
 1
 0.73 (0.5-1.0)

## Slide 4
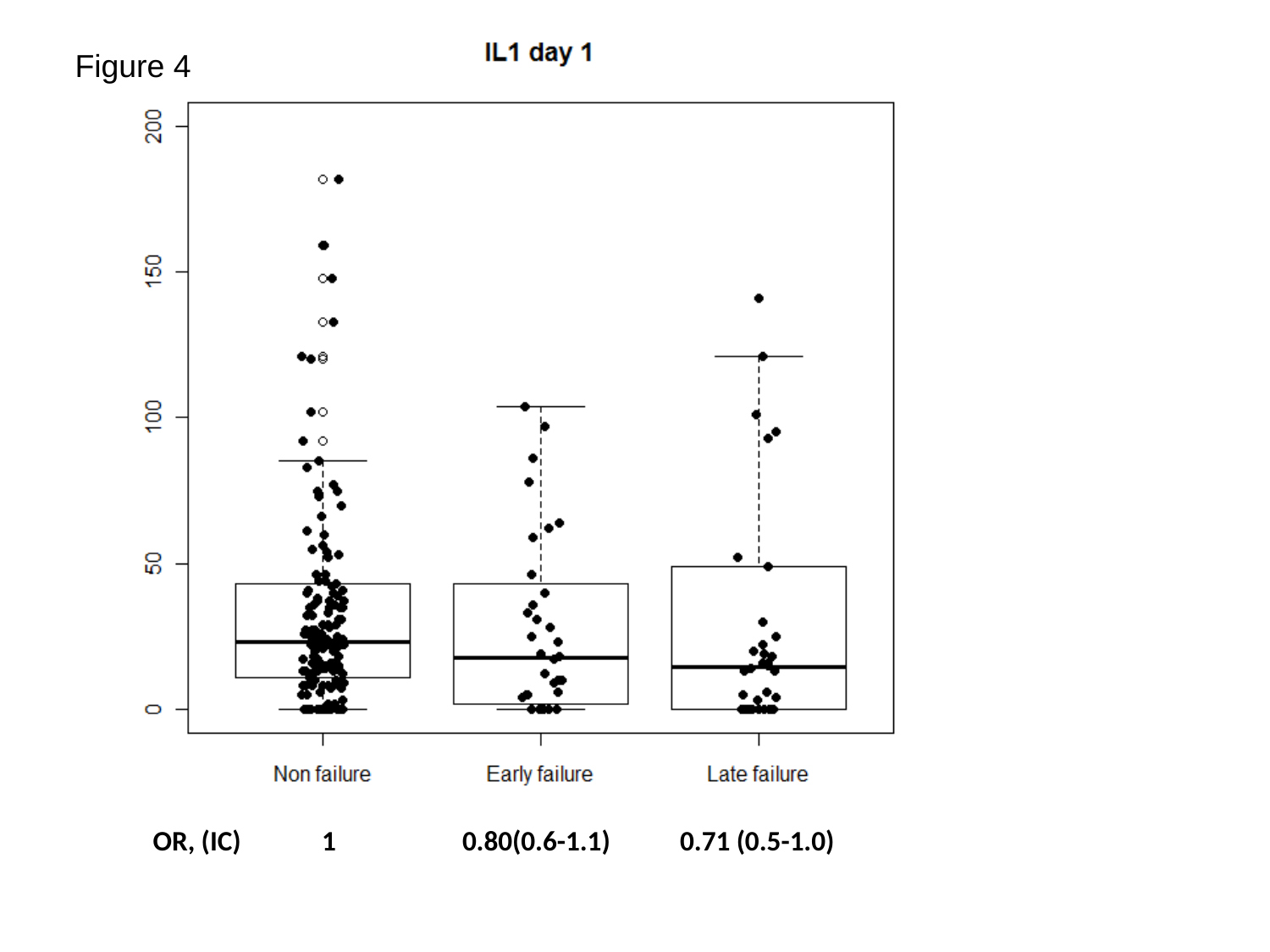

Figure 4
OR, (IC)
 1
0.80(0.6-1.1) 0.71 (0.5-1.0)

## Slide 5
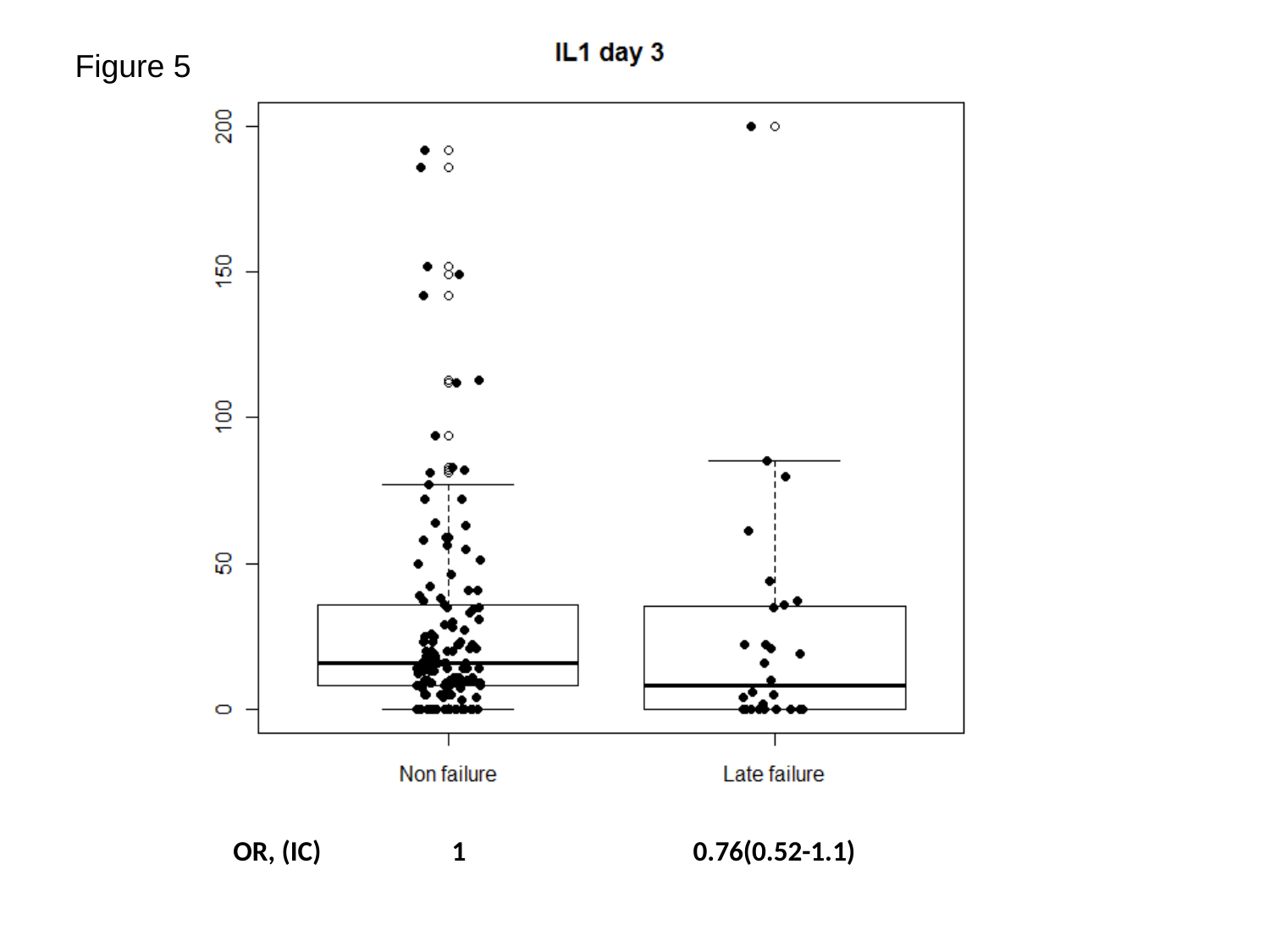

Figure 5
OR, (IC)
 1
0.76(0.52-1.1)

## Slide 6
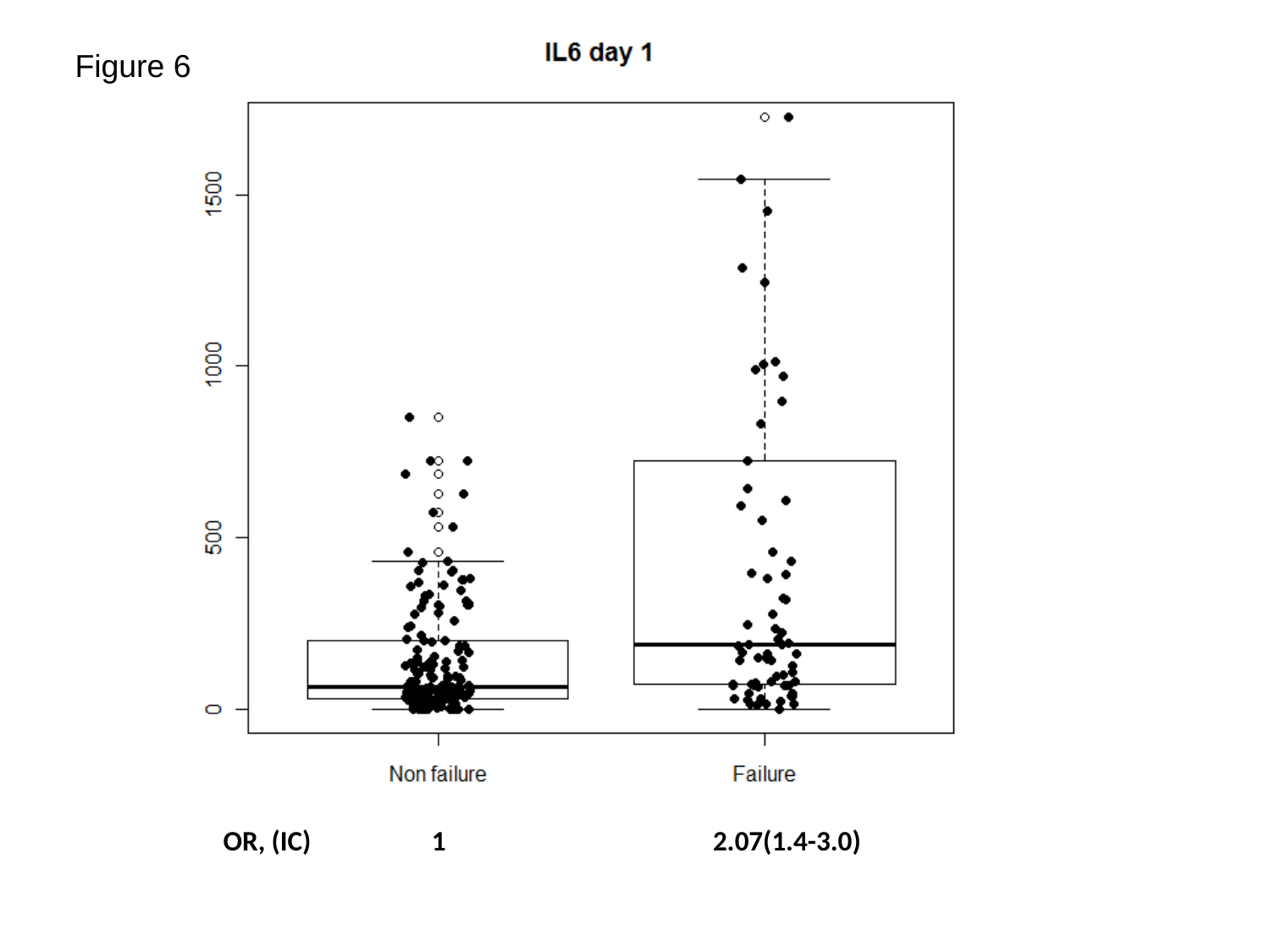

Figure 6
OR, (IC)
 1
2.07(1.4-3.0)

## Slide 7
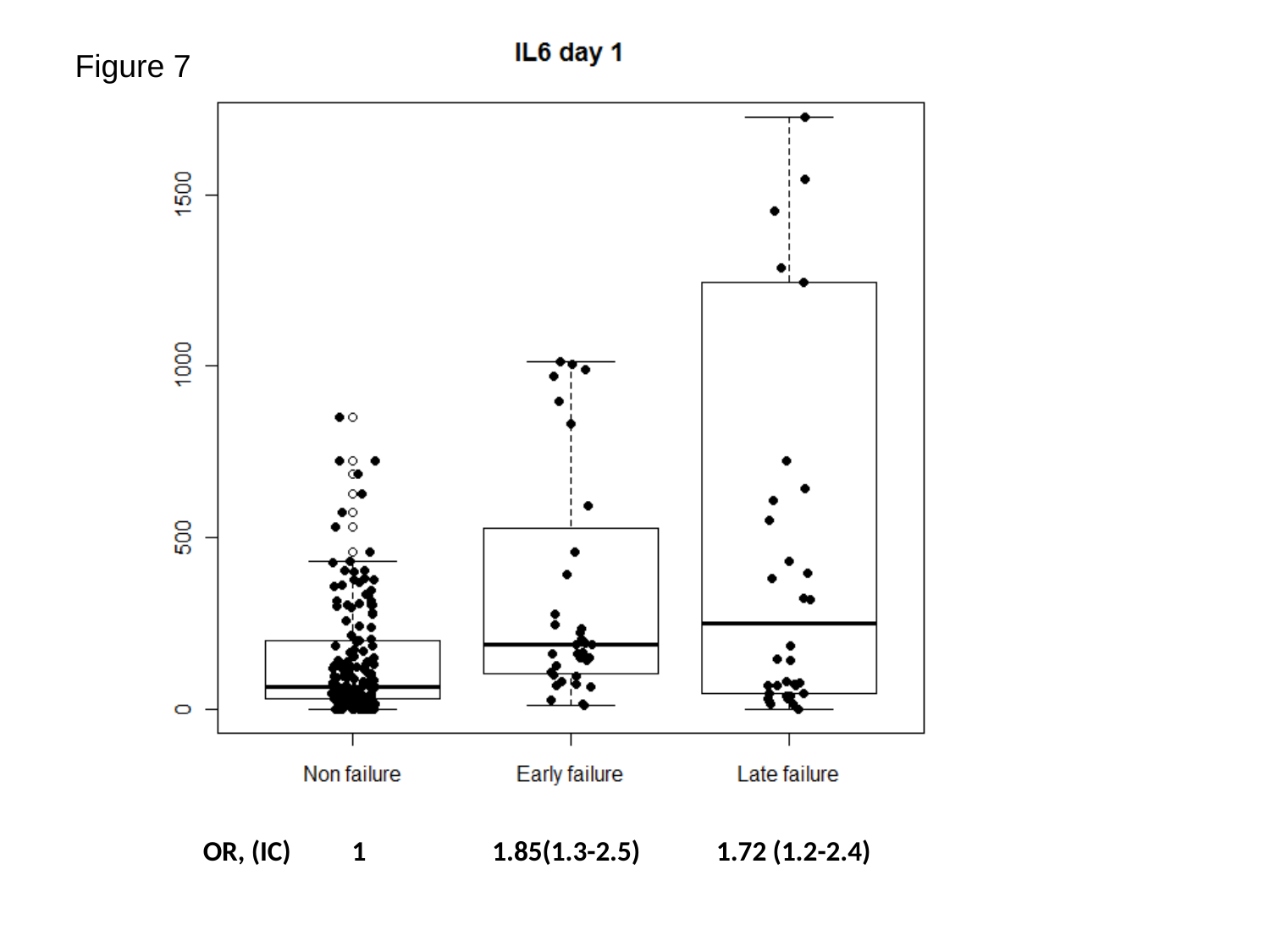

Figure 7
OR, (IC)
 1
1.85(1.3-2.5) 1.72 (1.2-2.4)

## Slide 8
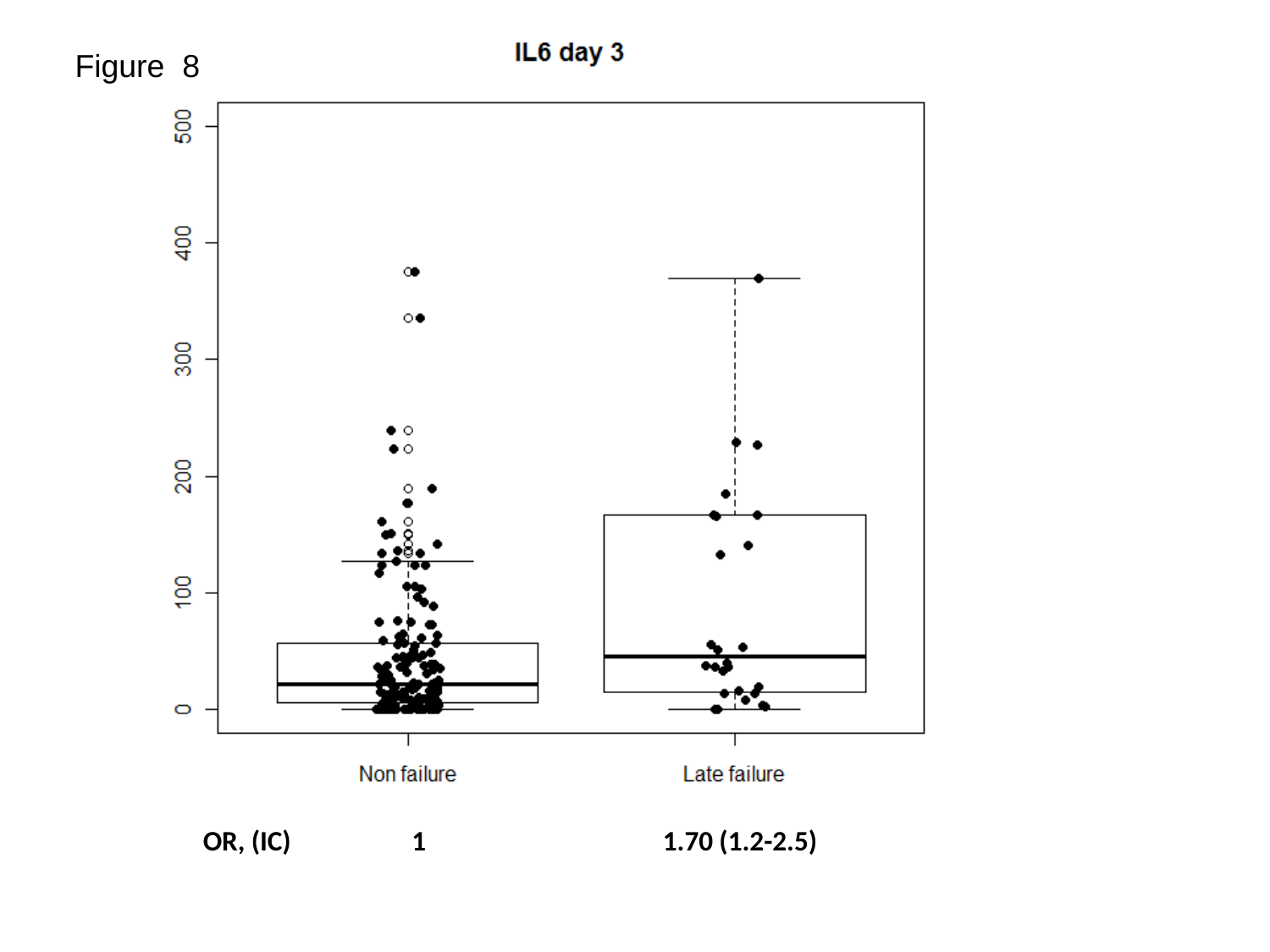

Figure 8
OR, (IC)
 1
1.70 (1.2-2.5)

## Slide 9
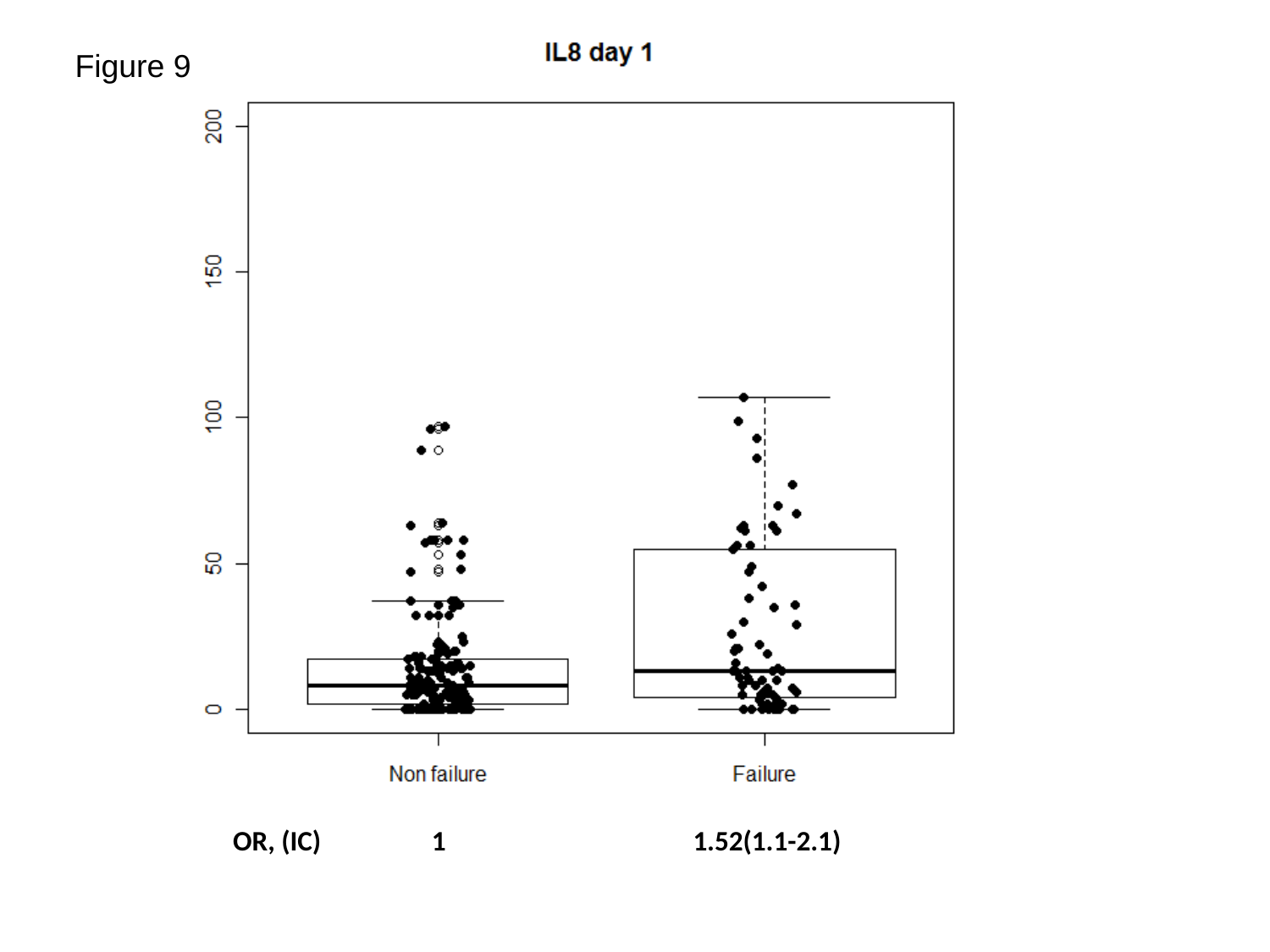

Figure 9
OR, (IC)
 1
1.52(1.1-2.1)

## Slide 10
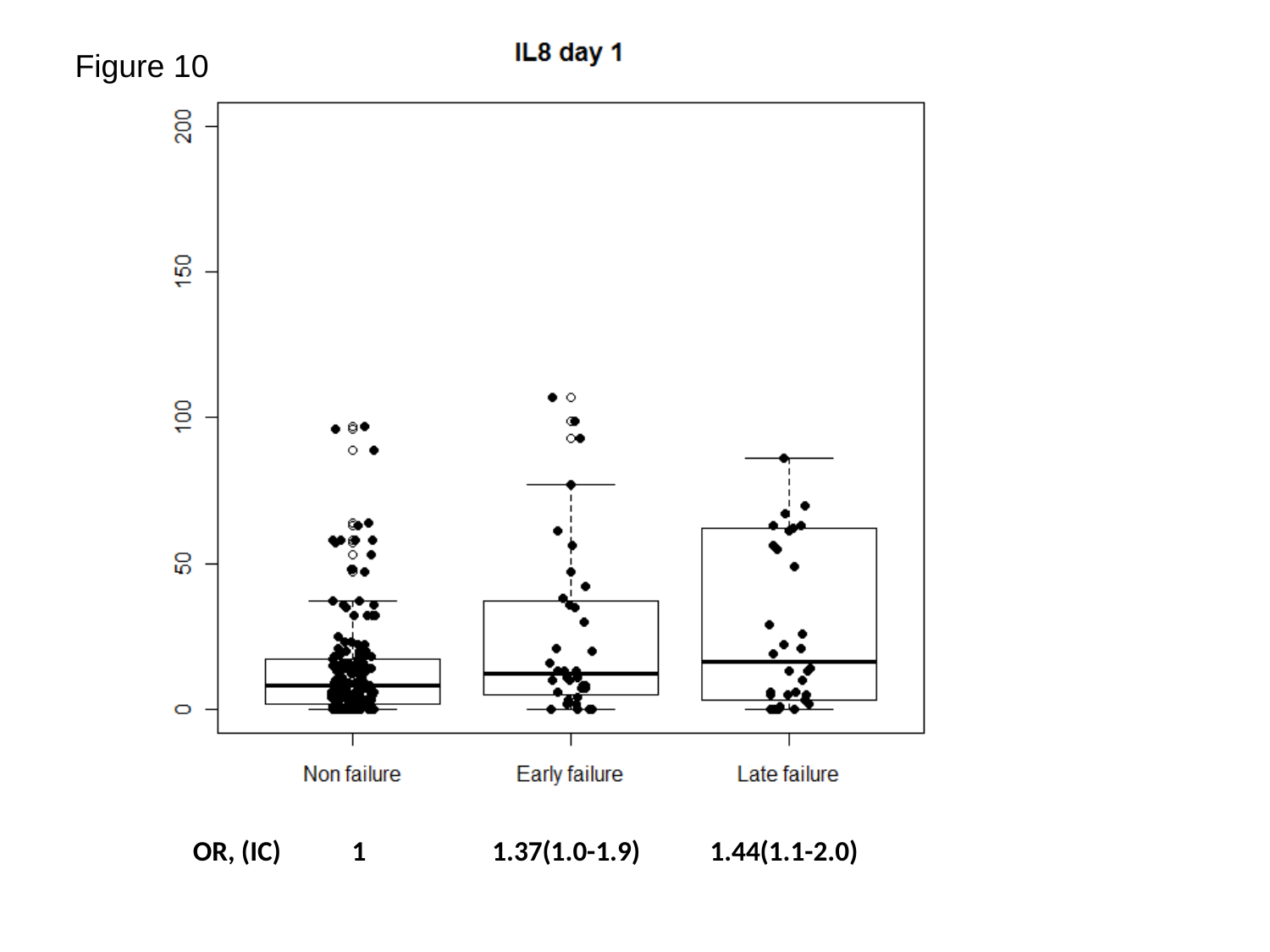

Figure 10
OR, (IC)
 1
1.37(1.0-1.9) 1.44(1.1-2.0)
